# Addressing the Silent Pandemic: The Role of Italian Primary Care Paediatrics in Combating Antimicrobial Resistance

**DOI:** 10.1111/apa.70380

**Published:** 2025-11-22

**Authors:** Giovanni Cerimoniale, Elena Chiappini, Nicolò Monti, Antonio D'Avino, Nicola Roberto Caputo, Osama Al Jamal, Giuseppe Di Mauro, Domenico Careddu, Antonella Antonelli, Silvia Zecca, Paolo Felice, Raffaele Losco

**Affiliations:** ^1^ FIMP (Italian Federation of Pediatricians), Member of the National PN‐CAR Ministerial Committee Latina Italy; ^2^ Department of Health Sciences University of Florence Florence Italy; ^3^ Infectious Diseases Unit Meyer Children's Hospital IRCCS Florence Italy; ^4^ Postgraduate School of Pediatrics, Department of Health Sciences University of Florence Florence Italy; ^5^ FIMP Naples Italy; ^6^ FIMP Brescia Italy; ^7^ FIMP Cagliari Italy; ^8^ FIMP Caserta Italy; ^9^ FIMP Novara Italy; ^10^ FIMP Rimini Italy; ^11^ FIMP Savona Italy; ^12^ FIMP Agrigento Italy; ^13^ FIMP Board of Probiviri Naples Italy

## 1 Background

The discovery of penicillin by Alexander Fleming in 1928 marked the beginning of the antibiotic era [1]. Since then, the development of antimicrobial agents has transformed modern medicine, yet each new antibiotic has been accompanied by the inevitable emergence of bacterial resistance [1]. In response to this threat, the Infectious Diseases Society of America (IDSA) formally introduced in 2007 the concept of antimicrobial stewardship programs (ASPs), defined as a coordinated set of interventions aimed at optimising antimicrobial use—choosing the right agent, dose, route and duration—without compromising patient outcomes [1, 2].

The Italian Ministry of Health launched the first National Action Plan on Antimicrobial Resistance (PNCAR) in 2017, establishing a multisectoral task force that included family paediatricians, thereby recognising the key role of primary care in antimicrobial resistance (AMR) containment.

In Italy, AMR is estimated to cause over 11 000 deaths each year—accounting for one‐third of all AMR‐related deaths in Europe, with a significant proportion occurring in younger individuals [2].

In this context, family paediatricians are pivotal. Their trusted relationships with families position them as central figures in promoting judicious antibiotic use and raising public awareness about infection prevention.

The most recent *AIFA National Report on Antibiotic Use in Italy* (2023) underscores critical and persistent issues in paediatric prescribing, placing renewed responsibility on primary care paediatricians [3]. In 2023, 40.9% of children aged 0–13 years received at least one systemic antibiotic—up from 33.7% in 2022. Many of these prescriptions were deemed inappropriate, particularly among children aged 2–5 years, in whom viral infections are the most common cause of fever and respiratory symptoms [3]. There has also been a notable decline in adherence to clinical guidelines, coupled with increased use of broad‐spectrum antibiotics. Only 54.4% of prescriptions were from the WHO ‘Access’ group, well below the EU's 65% target [2, 4].

Significant regional disparities persist; in Northern Italy, 37.7% of children receive antibiotics, compared to 44.1% in the South [4]. Moreover, broad‐spectrum antibiotics such as macrolides and third‐generation cephalosporins are heavily overused in southern regions, while narrower‐spectrum penicillin remains more common in the North [4]. The amoxicillin/amoxicillin‐clavulanate ratio—an established quality indicator—is 0.61 in the North but just 0.20 in the South [4]. Even more striking is the broad‐to‐narrow spectrum antibiotic ratio: 10.9 in the South versus 2.8 in the North, highlighting how Italy continues to lag behind European benchmarks [4]. These findings cast serious doubt on whether the PNCAR 2022–2025 target of a ≥ 20% reduction in this ratio can be met [4].

A prospective observational study in Italy (Picca et al.) between 2019 and 2021 examined antibiotic prescribing practices among family paediatricians for children with respiratory tract infections. Amoxicillin‐clavulanate emerged as the most frequently prescribed agent, and bronchitis—typically viral—accounted for 73% of inappropriate prescriptions. Fever alone tripled the likelihood of antibiotic use, even in cases not requiring it [3]. These findings align with the 2023 AIFA report, which recorded a 5.4% increase in systemic antibiotic use compared to 2022, undermining the PNCAR goal of a 10% reduction by 2025 [4].

An emerging concern is the growing role of *Service Pharmacies*, introduced by Decree Law No. 73 (June 7, 2024). While they offer rapid diagnostic testing (e.g., SARS‐CoV‐2 and group A streptococcus antigen detection, CRP), the lack of physician oversight often leads to antibiotic prescriptions based solely on test results, bypassing essential clinical judgement.

With nearly 90% of antibiotic prescriptions originating in primary care, the PNCAR 2022–2025 has introduced targeted interventions to curb inappropriate use. A *Territorial Antimicrobial Stewardship Program* has been launched, focusing on:

1. surveillance of antibiotic use and resistance trends;
2. dissemination of diagnostic and treatment protocols for common paediatric infections;
3. use of rapid diagnostics and biomarkers (e.g., CRP, RADT) in community settings and
4. continuous education for paediatricians on evidence‐based prescribing and clinical decision‐making.

These interventions aim to align community prescribing practices with national and global AMR containment goals [4] and funds from the National Recovery and Resilience Plan (NRRP) were allocated—as established by Ministerial Decree No. 226 of 29 July 2022—to support the purchase of diagnostic equipment for General Practitioners (GPs) and Family Paediatricians. However, these funds have not yet been used.

On March 27, 2025, the Italian Ministry of Health submitted for approval the draft agreement between the Government, Regions and Autonomous Provinces to allocate €120 million for the implementation of the PNCAR 2022–2025 (€40 million per year from 2023 to 2025). This investment will support surveillance, prevention and appropriate antibiotic use through a cross‐sectoral approach aligned with the WHO's One Health framework, integrating human, veterinary and environmental health.

The *ECDC Annual Epidemiological Report 2023* provides a sobering overview of AMR in Europe, with Italy among the most affected nations. Southern and southeastern Europe report the highest incidence of bloodstream infections caused by multidrug‐resistant organisms. In Italy, carbapenem‐resistant *Klebsiella pneumoniae* , MRSA and vancomycin‐resistant *Enterococcus faecium* (VREfm) are of particular concern, with VREfm prevalence exceeding 20%. Alarming levels of carbapenem resistance are also reported in *Pseudomonas aeruginosa* and *Acinetobacter baumannii* [2, 4].

Europe faces approximately 25 000 AMR‐related deaths annually, with healthcare and productivity costs nearing €1.5 billion [4]. Meanwhile, in the United States, outpatient antibiotic prescribing remains widespread, particularly among young children, who receive over 65 million prescriptions annually—at least 29% of which are unnecessary [5].

## 2 A Practical Survey

### 2.1 Method

To evaluate the appropriateness of antibiotic prescribing among family paediatricians in Italy, an online survey was conducted among 601 paediatricians [(601/6706 family paediatrician active in Italy; 9%) [6]] enrolled across regions of the country. All family paediatricians affiliated with FIMP (Italian Federation of Pediatricians) were recruited through an informative email regarding the study objectives and design. Each paediatrician could voluntarily and freely access the questionnaire, and answers were anonymously recorded. Participants were asked which antibiotic and treatment duration they would prescribe in three clinical scenarios: pharyngitis with a positive rapid antigen detection test (RADT) for group A Streptococcus (GABHS), acute otitis media (AOM) and community‐acquired pneumonia (CAP). Data were subsequently entered into an electronic database and analysed.

### 2.2 Result

**FIGURE 1:**
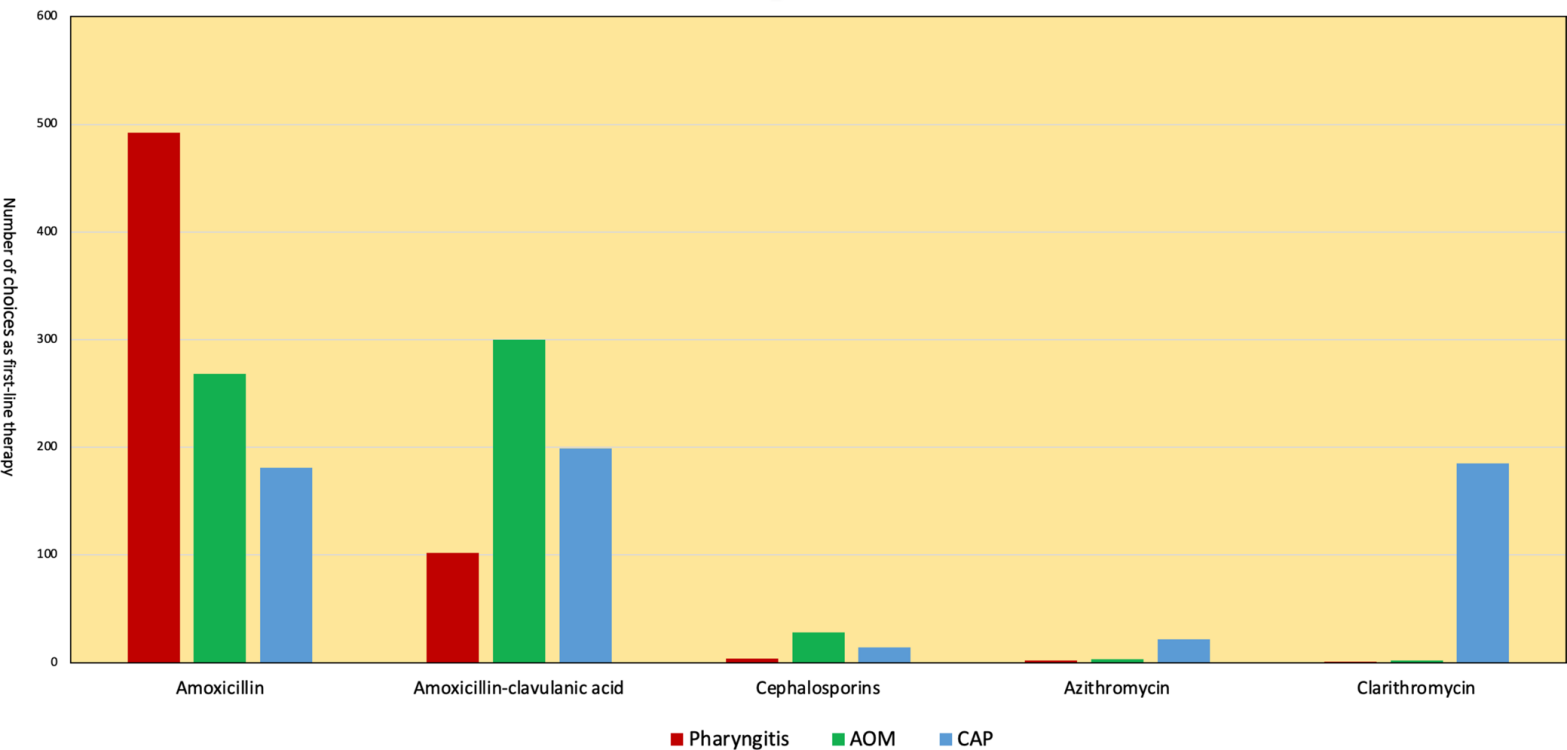
Distribution of antibiotic choices in the management of pharyngitis, acute otitis media and community‐acquired pneumonia by 601 paediatricians in Italy. Pharyngitis: pharyngitis with a positive rapid antigen detection test (RADT) for group A β‐haemolytic streptococcus (GABHS). AOM, acute otitis media; CAP, community‐acquired pneumonia.

For GABHS pharyngitis, 81.9% of physicians prescribed amoxicillin, but 16.7% used amoxicillin‐clavulanate (Figure 1). A 10‐day course was most frequent (63.9%), although 31.8% opted for 6 days. In AOM, amoxicillin‐clavulanate was the most prescribed antibiotic (49.9%), followed by amoxicillin (44.6%) (Figure 1); treatment lasted ≥ 6 days in over 90% of cases. For CAP, amoxicillin‐clavulanate (33.1%), amoxicillin (30.1%) and macrolides (34.5%) were most frequently prescribed (Figure 1). Treatment was prolonged in most cases, with 62.1% prescribing 10 days and 15% up to 15 days.

### 2.3 Discussion

Our study reveals a persistently widespread use of amoxicillin‐clavulanate for both GABHS pharyngitis and acute otitis media (Figure 1). A recent Italian intersociety consensus (2024) [7] recommended amoxicillin as the first‐choice antibiotic for children with GABHS pharyngotonsillitis (Table 1). Given the lack of robust evidence, the recommendation for a 10‐day course of therapy is conservatively maintained, particularly considering the increased risk of acute rheumatic fever in several Italian regions.

**TABLE 1:** Antibiotic therapy to be administered in children in cases of pharyngitis with a positive RADT for group A streptococcus, acute otitis media and mild–moderate community‐acquired pneumonia according to the Italian intersociety consensus (SIPPS‐SIP‐SITIP‐FIMP‐SIAIP‐SIMRI‐FIMMG‐ SIMG) [7, 8, 9].

Type of infection	Antibiotic therapy recommendation	Duration of treatment
Pharyngitis with a positive RADT for group A streptococcus	*Amoxicillin 50 mg/kg/day divided into two administrations may be recommended* In patients with suspected penicillin allergy the alternative antibiotic regimen are third‐generation cephalosporin or macrolides [7]	10 days 5 days with third‐generation cephalosporin or macrolides
Acute otitis media (AOM)	Children aged 6–24 months with mild unilateral AOM and children older than 24 months with unilateral or bilateral AOM: a watchful waiting strategy should be recommended^a^ Infants < 6 months and children aged 6–24 months with bilateral AOM: immediate antibiotic therapy Children with otorrhoea, severe bilateral AOM, AOM with systemic symptoms and impaired general status [8, 10] *1st choice: Amoxicillin 80–90 mg/kg/day in three divided doses*; 2nd choice: Amoxicillin +clavulanic acid 80–90 mg/kg/day in two divided doses [8, 10] Cefuroxime or cefpodoxime proxetil is recommended for patients with suspected allergy to amoxicillin at low risk of allergic reaction. The use of macrolides or clindamycin should be reserved for patients at high risk of allergic reactions [8, 10]	7 or 10 days in high‐risk or severe conditions For children without risk factors for unfavourable outcomes^b^ a shorter course of 5 days
Mild–moderate Community acquired Pneumonia (CAP)	*Amoxicillin 80–90 mg/kg/day divided into three separate doses* [9] For children who are either unimmunized or have incomplete immunisation coverage for both *H. influenzae* type b and *S. pneumoniae* (having received less than two doses of hexavalent and pneumococcal vaccines), first‐line therapy with amoxicillin‐clavulanate or second‐ or third‐generation cephalosporins is recommended [9] Cefuroxime or cefpodoxime proxetil is recommended for patients with suspected allergy to amoxicillin at low risk of allergic reaction. The utilisation of macrolides or clindamycin should be reserved for patients at high risk of allergic reaction, with consideration given to levofloxacin for children > 12 years [9]	5‐days If necessary, treatment may be extended for up to 7 days

^a^ A watchful waiting strategy is recommended in all cases without comorbidities or risk factors and considering specific severity conditions requiring immediate treatment.

^b^ Older than 2 years, without otorrhoea, without recurrence, without bilaterality, without severe symptoms, after a period of watchful waiting.

Both the *National Institute for Health and Care Excellence* (NICE) committee and the Italian Society of Paediatrics (SIP) guidelines suggest that amoxicillin remains the first‐choice antibiotic for treating AOM in children, as it is widely used and has an acceptable resistance profile [8, 10]. The SIP guidelines further recommend extending amoxicillin therapy to 10 days in children at risk of poor outcomes (e.g., those under 2 years of age or presenting with spontaneous otorrhoea) [8, 10] (Table 1). In our study, macrolides were the first‐choice molecules in children with community‐acquired pneumonia, followed by amoxicillin‐clavulanate (Figure 1). As reported in the recent Italian intersociety consensus (2024), in children with a complete immunisation schedule for *Streptococcus pneumoniae* and/or *Haemophilus influenzae* and mild–moderate CAP, amoxicillin is the first‐choice drug [9]. Moreover, in our survey, over 75% of paediatricians reported prescribing treatment durations of 10 days or more for mild to moderate CAP. However, according to current guidelines [9], a 5‐day course of antibiotic therapy with amoxicillin is recommended. Close clinical monitoring and reassessment are advised approximately 72 h after initiating antibiotic therapy to evaluate symptom resolution. If necessary, treatment may be extended to a total of 7 days [9] (Table 1). The study reveals that the first‐line therapeutic approach to the three most common infections encountered by primary care paediatricians diverges from both national and international recommendations, in terms of both antibiotic selection and treatment duration (Table 1). These prescribing patterns may contribute to rising AMR, as evidenced by the increased resistance of *S. pneumoniae* to macrolides highlighted in the 2023 AIFA report, which documented a 26.3% rise in invasive pneumococcal infections and growing resistance to clarithromycin and azithromycin [4]. This evidence underscores the urgency of aligning clinical practice with current guidelines to curb AMR and ensure effective paediatric care in Italy.

## 3 Conclusion

AMR remains a major public health concern, with inappropriate antibiotic prescribing in Italian paediatric primary care contributing significantly to the problem. Despite national guidelines and stewardship efforts, the overuse of broad‐spectrum antibiotics—particularly amoxicillin‐clavulanate and macrolides—persists, along with unnecessarily long treatment durations. Our survey confirms these critical gaps, especially in managing common respiratory tract infections and regional disparities further highlight the need for targeted interventions. To meet national and international AMR containment goals, it is essential to align paediatric prescribing with current evidence, strengthen territorial stewardship programs, and support family paediatricians through education, surveillance and diagnostic tools. Strengthening these interventions is crucial to align prescribing behaviours with current guidelines, reduce regional disparities and curb the rise of antimicrobial resistance.

## Author Contributions

G.C. contributed to the conceptualization and study design. G.C., E.C., N.M., A.D., N.R.C., O.A.J., G.D.M., D.C., A.A., S.Z., P.F. and R.L. contributed to data collection, patient management, and analysis. E.C., and N.M. drafted the manuscript, and all authors critically reviewed and approved the final version.

## Funding

The authors have nothing to report.

## Ethics Statement

This study was conducted in accordance with the ethical principles outlined in the Declaration of Helsinki.

## Consent

The authors have nothing to report.

## Conflicts of Interest

The authors declare no conflicts of interest.

## Data Availability

The data that support the findings of this study are available on request from the corresponding author. The data are not publicly available due to privacy or ethical restrictions.
